# Uremic Toxins Inhibit Transport by Breast Cancer Resistance Protein and Multidrug Resistance Protein 4 at Clinically Relevant Concentrations

**DOI:** 10.1371/journal.pone.0018438

**Published:** 2011-04-04

**Authors:** Henricus A. M. Mutsaers, Lambertus P. van den Heuvel, Lauke H. J. Ringens, Anita C. A. Dankers, Frans G. M. Russel, Jack F. M. Wetzels, Joost G. Hoenderop, Rosalinde Masereeuw

**Affiliations:** 1 Department of Pharmacology and Toxicology, Radboud University Nijmegen Medical Centre, Nijmegen Centre for Molecular Life Sciences, Nijmegen, The Netherlands; 2 Department of Physiology, Radboud University Nijmegen Medical Centre, Nijmegen Centre for Molecular Life Sciences, Nijmegen, The Netherlands; 3 Department of Pediatrics, Radboud University Nijmegen Medical Centre, Nijmegen, The Netherlands; 4 Department of Nephrology, Radboud University Nijmegen Medical Centre, Nijmegen, The Netherlands; 5 Department of Pediatrics, Catholic University Leuven, Leuven, Belgium; University of Cambridge, United Kingdom

## Abstract

During chronic kidney disease (CKD), there is a progressive accumulation of toxic solutes due to inadequate renal clearance. Here, the interaction between uremic toxins and two important efflux pumps, *viz.* multidrug resistance protein 4 (MRP4) and breast cancer resistance protein (BCRP) was investigated. Membrane vesicles isolated from MRP4- or BCRP-overexpressing human embryonic kidney cells were used to study the impact of uremic toxins on substrate specific uptake. Furthermore, the concentrations of various uremic toxins were determined in plasma of CKD patients using high performance liquid chromatography and liquid chromatography/tandem mass spectrometry. Our results show that hippuric acid, indoxyl sulfate and kynurenic acid inhibit MRP4-mediated [^3^H]-methotrexate ([^3^H]-MTX) uptake (calculated Ki values: 2.5 mM, 1 mM, 25 µM, respectively) and BCRP-mediated [^3^H]-estrone sulfate ([^3^H]-E1S) uptake (Ki values: 4 mM, 500 µM and 50 µM, respectively), whereas indole-3-acetic acid and phenylacetic acid reduce [^3^H]-MTX uptake by MRP4 only (Ki value: 2 mM and IC_50_ value: 7 mM, respectively). In contrast, p-cresol, p-toluenesulfonic acid, putrescine, oxalate and quinolinic acid did not alter transport mediated by MRP4 or BCRP. In addition, our results show that hippuric acid, indole-3-acetic acid, indoxyl sulfate, kynurenic acid and phenylacetic acid accumulate in plasma of end-stage CKD patients with mean concentrations of 160 µM, 4 µM, 129 µM, 1 µM and 18 µM, respectively. Moreover, calculated Ki values are below the maximal plasma concentrations of the tested toxins. In conclusion, this study shows that several uremic toxins inhibit active transport by MRP4 and BCRP at clinically relevant concentrations.

## Introduction

Approximately 5% of the adult population in the developed countries suffers from chronic kidney disease (CKD) stage III-V, which is defined by a decreased estimated glomerular filtration rate (eGFR) [1]. A main feature at this stage of CKD is the accumulation of solutes that are normally excreted in urine. These uremic retention solutes, also known as uremic toxins, are a heterogeneous group of organic compounds. Currently, 110 compounds are considered to be uremic toxins and they are classified into three groups depending on their chemical properties that largely influence the possibility to remove these toxins using current dialysis strategies, namely size and solubility. The currently defined groups, as described by Vanholder *et al.* 2008, are: (1) the small water-soluble compounds, with a molecular weight (MW) arbitrarily set at ≤500 Da, for example, urea and creatinine; these compounds are easily removed via dialysis and their toxic potential is limited. (2) The middle molecules, with a MW >500 Da, such as β_2_-microglobulin; due to their size, these retention solutes can only be cleared using dialyzer membranes with large pores, which focus on filtration via convection instead of diffusion. (3) The protein-bound solutes; the compounds in this group mostly have a small MW and prototypes include indoxyl sulfate and p-cresol sulfate. Solutes belonging to this group are very difficult to clear using current dialysis strategies and they exhibit toxic effects [2]. Uremic toxins are thought to contribute to the plethora of pathologies observed in patients with CKD, including anemia, bone disorders, renal fibrosis and cardio-vascular disease. Administration of the oral sorbent AST-120 is currently the only therapy to prevent accumulation of protein-bound uremic toxins in patients with CKD. Unfortunately, AST-120 prevents no more than the uptake of indoxyl sulfate and p-cresol in the intestinal tract [3]. Understanding the endogenous clearance of protein-bound solutes could lead to the development of novel therapeutic strategies for the removal of uremic toxins.

In the healthy population, uremic toxins are cleared by the kidney and this process is largely dependent on glomerular filtration and tubular secretion via a multitude of transport proteins expressed in renal proximal tubules. Moreover, it has been demonstrated that both organic anion transporter (OAT) 1 and OAT3 play important roles in the renal tubular uptake of uremic toxins and organic anions [4]–[6]. Both transporters show overlapping substrate specificities, but differential contributions to uremic toxin clearance have been reported as well. For example, indoxyl sulfate is equally transported by OAT1 and OAT3, but indole-3-acetic acid and hippuric acid are preferable substrates for OAT1, and uptake of 3-carboxy-4-methyl-5-propyl-2-furanpropionate is mediated by OAT3 solely [6]. In addition, using a rat model of renal failure, the basolaterally expressed kidney-specific organic anion transporting polypeptide 4C1 (SLCO4C1) was recently shown to facilitate the removal of several uremic toxins, including guanidino succinate, in the proximal tubule [7]. Thus, basolateral uptake of uremic toxins in renal proximal tubules cells is fairly well characterized, however, little is known about the transport of uremic toxins over the apical membrane into urine. Two important renal efflux pumps at the apical membrane are multidrug resistance protein 4 (MRP4) and breast cancer resistance protein (BCRP) [8], [9]. Both MRP4 and BCRP are known to transport urate, [10], [11] a uremic toxin involved in the pathogenesis of gout and cardiovascular disease [12]. Furthermore, functional and nonfunctional mutations in the BCRP gene cause hyperuricemia-based gout, supporting the importance of the efflux pump in urate secretion [11], [13]. Therefore, it seems likely that both MRP4 and BCRP are involved in the transport of uremic toxins into the proximal tubule lumen.

The present study was designed to investigate the interaction between several uremic toxins, mainly belonging to the group of protein-bound solutes, and MRP4- and BCRP-mediated transport. Our results show that hippuric acid, indoxyl sulfate and kynurenic acid inhibit substrate specific uptake by both MRP4 and BCRP, whereas indole-3-acetic acid and phenylacetic acid only reduce transport by MRP4. Moreover, inhibition of transport by multiple uremic toxins mainly occurs at clinically relevant concentrations, suggesting that uremic toxins may contribute to the many complications of CKD.

## Materials and Methods

### Ethics Statement

The ethical committee of the Radboud University Nijmegen Medical Centre on research involving human subjects approved this study, and oral informed consent was obtained from each patient and each healthy volunteer.

### Chemicals

All chemicals were obtained from Sigma (Zwijndrecht, the Netherlands) unless stated otherwise. Stock solutions of uremic toxins were prepared as previously described [14], and stored at −20 °C. [3′,5′,7′-^3^H(*n*)]-methotrexate disodium salt ([^3^H]-MTX) with a specific activity ranging between 13.4 and 25.3 Ci/mmol was purchased from Moravek (Brea, USA) and [6′,7′-^3^H(*n*)]-estrone-sulfate ammonium salt ([^3^H]-E1S) with a specific activity of 54.26 Ci/mmol was obtained from Perkin Elmer (Groningen, the Netherlands).

### Cell culture and transfection

Human embryonic kidney (HEK293; purchased at American Type Culture Collection, Manassas, VA) cells were cultured in Dulbecco's modified Eagle's medium (Invitrogen life sciences, Breda, the Netherlands) containing 10% (v/v) fetal calf serum (MP Biomedicals, Uden, the Netherlands) at 37°C in a 5% (v/v) CO_2_ atmosphere. To functionally overexpress MRP4 and BCRP, HEK293 cells were transduced with baculoviruses of human MRP4, BCRP or enhanced yellow fluorescent protein (EYFP), generated via the Bac-to-Bac system (Invitrogen) as previously described [15]. To transduce HEK293 cells, they were cultured in 500 cm^2^ flasks until 70% confluence. Subsequently, medium was removed and 10 ml of virus and 25 ml of medium were added and incubated for 30 min at 37°C. Next, 50 ml of medium was added and after 2 h of transduction 5 mM sodium butyrate was added.

### Membrane vesicle preparation

Three days after transduction, cells were harvested and pelleted by centrifugation (30 min at 4,000 x g). Afterwards, the cells were resuspended in ice-cold hypotonic TS buffer (0.5 mM sodium phosphate, 0.1 mM EDTA, pH 7.0) containing protease inhibitors (100 µM phenylmethylsulfonyl fluoride, 5 µg/ml aprotinin, 5 µg/ml leupeptin, 1 µg/ml pepstatin and 1 µg/ml E-64) and shaken for 30 min at 4°C. Cells were then centrifuged at 100,000 x g for 30 min at 4°C. Subsequently, pellet was resuspended in ice-cold isotonic buffer (10 mM Tris-HEPES and 250 mM sucrose, pH 7.4, adjusted with HEPES) supplemented with protease inhibitors and homogenized using a tight fitting Dounce homogenizer followed by centrifugation (1,000 x g, 20 min, 4°C). Afterwards, supernatant was centrifuged at 100,000 x g for 1 h at 4°C. The resulting pellet was resuspended in isotonic buffer and passed through a 27-gauge needle 25 times to obtain crude membrane vesicles. The protein content of samples was determined using the Bio-Rad protein assay (Veenendaal, the Netherlands), according to manufacturers recommendations. Vesicles were frozen in liquid nitrogen and stored at −80°C until use. The orientation of the membrane vesicles was not determined, since ATP-dependent uptake occurs only in inside-out vesicles.

### Western blotting

Overexpression of MRP4 or BCRP in membrane vesicles was studied using the Odyssey western blotting technique. Total protein (15 µg) was separated via SDS/PAGE using a 10% (w/v) gel, and blotted onto nitrocellulose membranes using the iBlot dry blotting system (Invitrogen). Afterwards, the membrane was blocked using Odyssey Blocking Buffer, (1∶1 diluted with PBS; Westburg BV, Leusden, the Netherlands) for 1 hour at RT. The membrane was then incubated overnight at 4°C with rabbit-α-MRP4 (1∶5,000; van Aubel *et al.* [8]) or mouse-α-BCRP (1∶200; Clone BXP-21; Kamiya Biomedical, Seattle, USA) in Odyssey Blocking Buffer containing 0.1% (v/v) Tween-20. Afterwards, the membrane was thoroughly washed three times during 10 min with PBS containing 0.1% (v/v) Tween-20. The secondary antibodies, goat-α-rabbit IRDye 800 (1∶10,000; Sigma) and goat-α-mouse Alexa Fluor 680 (1∶10,000; Rockland, Heerhugowaard, the Netherlands), were incubated for 1 hour at RT in Odyssey Blocking Buffer containing 0.1% (v/v) Tween-20 and 0.01% (w/v) SDS. The membrane was thoroughly washed, as described above, and then scanned using the Odyssey Infrared Imaging System (LI-COR Biotechnology). Expression of MRP4 was assessed using channel 800 and BCRP expression was determined using channel 700.

### Membrane vesicle transport inhibition assay

A rapid filtration technique was used to study the uptake of [^3^H]-MTX and [^3^H]-E1S into MRP4 or BCRP membrane vesicles, as previously described [16]. In short, 25 µl of TS buffer containing 4 mM ATP, 10 mM MgCl_2_ and radiolabeled substrate was added to 5 µl of the membrane vesicles (1.5 mg/ml). The transport assay was performed in the absence or presence of various concentrations of uremic toxins to evaluate the inhibitory effects of these compounds on MRP4-mediated [^3^H]-MTX uptake and BCRP-mediated [^3^H]-E1S uptake. Transport was started by incubating the mixture at 37°C for 1 min (BCRP) or 10 min (MRP4), time points at which substrate uptake was previously shown to be linear [15], [17]. Uptake was stopped by placing the samples on ice and the addition of 150 µl ice cold TS buffer. Subsequently, the samples were transferred to a 96 well filter plate (Millipore, Etten-leur, the Netherlands) pre-incubated with TS buffer and filtered using a Multiscreen HTS-Vacuum Manifold filtration device (Millipore). Afterwards, 2 ml of scintillation liquid was added to each filter and radioactivity was determined using liquid scintillation counting. As negative controls ATP was substituted for AMP and EYFP-membrane vesicles were used. Each experiment was performed in triplicates.

### High-performance liquid chromatography (HPLC)

Blood samples were obtained from 4 patients with chronic renal failure (CRF) during regular check-up, 6 patients with end-stage renal disease (ESRD) before hemodialysis and 4 healthy controls. Clinical characteristics of study subjects are listed in Table 1. None of the subjects had been fasting at the time of blood sampling. Blood was collected in an EDTA Vacutainer and was immediately centrifuged at 3,000 x g for 10 min. Subsequently, plasma was collected and stored at −20°C. Before chromatography an aliquot of plasma was diluted in H_2_O (1∶1) and deproteinized with perchloric acid (final concentration 3.3% (v/v)). Next, samples were centrifuged at 12,000 x g for 3 min and 50 µl of the supernatant was injected into the HPLC-system (Spectra-Physics Analytical, Spectrasystem SCM400). To measure indole-3-acetic acid, indoxyl sulfate and hippuric acid, the HPLC was equipped with a C18 HPLC column (GraceSmart RP 18 5 u 150×4.6 mm; Grace, Breda, the Netherlands). Separation was performed at a flow rate of 1 ml/min with eluent A (95% (v/v) H_2_O, 5% (v/v) acetonitril and 0.1% (v/v) heptafluorobutyric acid) and eluent B (50% (v/v) H_2_O, 50% (v/v) acetonitrile and 0.1% (v/v) heptafluorobutyric acid) under the following gradient conditions: 0–1 min, 100% eluent A; 1–15 min, 100–25% eluent A; 15–17 min, 25% eluent A; 17–18 min, 0–100% eluent A; 18–23 min, 100% eluent A. The compounds were detected at a wavelength of 230 nm. For the detection of phenylacetic acid, chromatography was performed on a C18 HPLC column (Polaris 3 C18-A 150×4.6 mm; Varian, Middelburg, the Netherlands) with eluent A (97% (v/v) 50 mM sodium phosphate buffer [pH 6.5] and 3% (v/v) methanol) and eluent B (50% (v/v) H_2_O, 49% (v/v) acetonitrile and 1% (v/v) tetrahydrofuran) using the following gradient: 0–1 min, 100% eluent A; 1–15 min, 100–90% eluent A; 15–18 min, 10–100% eluent B; 18–22 min, 100% eluent B; 22–25 min, 0–100% eluent A; 25–30 min, 100% eluent A. The flow rate was 1 ml/min and phenylacetic acid was measured at a wavelength of 215 nm. Standards of the compounds were also run in order to quantify the amount of toxins found in the samples. Acquired data were processed with PC1000 software (Spectrasystem).

**Table 1:** Characteristics of study subjects.

	CRF	ESRD	Control
Number	4	6	4
Age (years)	54±20	58±13	34±10
Women (%)	25	33	25
Ureum (mmol/l)	32±6	21±6	ND
Creatinine (µmol/l)	510±210	720±90	ND
Dialysis strategy	NA	4 HD, 2 CAPD	NA

Values are shown as mean ± SD. CRF, chronic renal failure; ESRD, end-stage renal disease; ND, not determined; NA, not applicable; HD, hemodialysis; CAPD, continuous ambulatory peritoneal dialysis.

### Liquid chromatography/tandem mass spectrometry (LC/MS-MS)

To determine the levels of kynurenic acid, blood was collected from CRF patients and processed as described above. Subsequently, 10 µl of the clear supernatant was injected into the LC/MS-MS system that consisted of an Accela HPLC system (Thermo scientific, Breda, the Netherlands) equipped with a C18 HPLC column (VisionHT C18 B 100×2 mm, 1.5 µm; Grace). Separation was performed at a flow rate of 150 µl/min with eluent A (5 mM ammonium formate+0.01% (v/v) trifluoroacetic acid) and eluent B (50% acetonitrile) under the following gradient conditions: 0–10 min, 98–50% eluent A; 10–15 min, 50% eluent A; 15–16 min, 50–98% eluent A; 16–21 min, 98% eluent A. The fractions eluted were directly passed through a TSQ Vantage tandem mass spectrometer (Thermo scientific) equipped with an electro-spray ionization source operating in the positive ion mode. The ion spray voltage was 4 kV, source temperature was 350°C and collision gas pressure was 1.5 bar. Kynurinic acid and the internal standard 1-methyl-tryptophan were quantified by selected reaction monitoring (SRM). The following SRM transitions were used: *m/z* 190 (parent ion) to *m/z* 89 and 144 (both product ions) for kynurenic acid and *m/z* 219.1 (parent ion) to *m/z* 160 and 202.1 (product ions) for methyl-tryptophan. A calibration curve of kynurenic acid was made to quantify the amount of toxin found in the samples and the results were corrected using the internal standard. Acquired data were processed with Thermo Xcaliber software (Thermo scientific).

### Kinetic analysis and statistics

Statistics were performed using GraphPad Prism 5.02 via an unpaired t test or a Kruskal-Wallis test followed by a Dunn's Multiple Comparison test. Differences between groups were considered to be statistically significant when p<0.05. The software was also used to perform (non-)linear regression analysis, curve fitting details are summarized in Table S1 . The mean IC_50_ and IC_20_ values were calculated from the inhibition curves used for the Dixon analysis and to determine the inhibition constant (Ki) from the Dixon plots. Transport inhibition studies were performed in triplicate and repeated at least three times.

## Results

### Selection of uremic toxins

**Figure 1:**
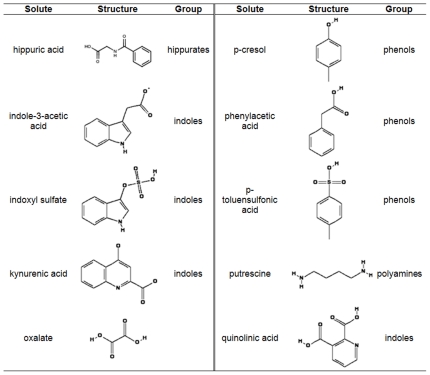
Selected uremic toxins. p-toluensulfonic acid was used as a model compound for p-cresol sulfate.

The number of solutes that are considered to be uremic toxins is constantly increasing [18]. As stated before, protein-bound toxins are difficult to eliminate via dialysis and, therefore, these toxins accumulate and become players in the multitude of pathologies observed in uremic patients. In our study, ten toxins were selected containing one water-soluble solute (oxalate) and nine protein-bound solutes. The latter group contained four indoles (indoxyl sulfate, indole-3-acetic acid, kynurenic acid and quinolinic acid), three phenols (phenylacetic acid, p-cresol and p-toluenesulfonic acid as a model compound for p-cresol sulfate), one hippurate (hippuric acid) and one polyamine (putrescine). Chemical characteristics of the solutes studied are depicted in Figure 1.

### Expression of MRP4 and BCRP, and uptake of [^3^H]-MTX and [^3^H]-E1S by membrane vesicles

**Figure 2:**
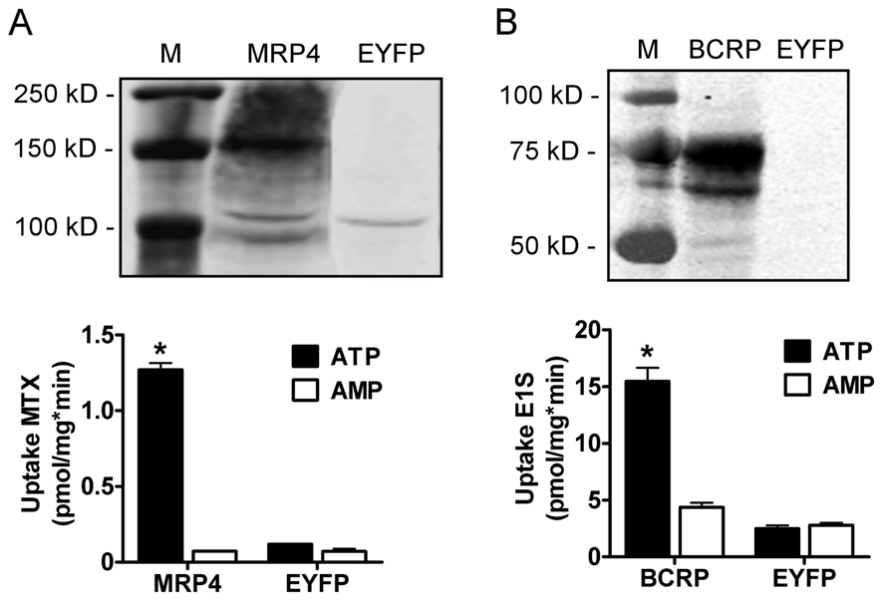
Transport expression and activity in isolated membrane vesicles. HEK293 cells were transfected with a baculovirus of either human MRP4 or BCRP. Cells transfected with EYFP were used as a negative control. Subsequently, membranes were isolated and vesicles were prepared. Proteins were separated via SDS/PAGE and blotted onto nitrocellulose membranes. Blots were incubated with an antibody against MRP4 or BCRP and the appropriated fluorescently labeled secondary antibodies. MRP4 was detected at 150 kD **(upper panel A)** and BCRP was detected at 75 kD **(upper panel B)**. A rapid filtration technique was used to study the ATP-dependent uptake of [^3^H]-MTX into MRP4- overexpressing membrane vesicles **(lower panel A)** and [^3^H]-E1S into BCRP membrane vesicles **(lower panel B)**. As negative control ATP was substituted for AMP. Results are presented as mean ± SEM of one representative experiment performed in triplicate. M  =  marker, *  =  p<0.0001 compared to EYFP. Experiments were performed at least three times.

Human embryonic kidney (HEK293) cells were transduced using a baculovirus system to overexpress human MRP4, BCRP or enhanced yellow fluorescent protein (EYFP; negative control). Using the Odyssey Western blot technique, MRP4 and BCRP were detected in membrane vesicles isolated from MRP4- and BCRP-overexpressing HEK293 cells at 150 kD and 75 kD, respectively. Protein expression of the transporters was absent in EYFP-transduced cells, indicating that endogenous expression was undetectable. For functionality of MRP4, the uptake of methotrexate (MTX) was investigated by using a radiotracer of the drug [16]. Figure 2A shows that the ATP-dependent uptake of [^3^H]-MTX in MRP4-overexpressing vesicles is 11-fold higher as compared to EYFP vesicles with an average rate of 1.3 pmol/mg*min. To determine the transport activity of BCRP, radioactively labeled estrone sulfate (E1S) was used as typical substrate [17]. BCRP-overexpressing vesicles showed 24-fold higher ATP-dependent uptake of [^3^H]-E1S, compared to EYFP controls with an average rate of 15.5 pmol/mg*min (Figure 2B). Furthermore, Figure 2 demonstrates that for both transporters non-specific, AMP-dependent, uptake is very low. Therefore, AMP-corrected uptake is shown in subsequent figures. These results support the functional expression of the transporters.

### Uremic toxins inhibit transport

**Figure 3:**
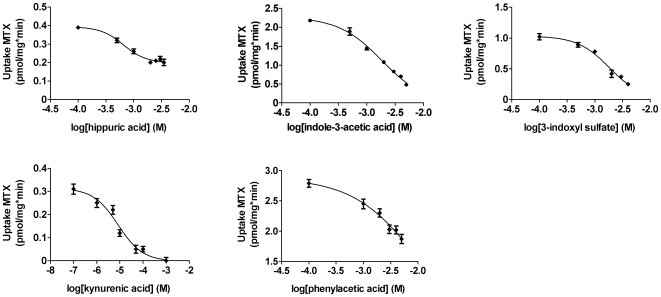
Uremic toxins inhibit MRP4-mediated transport. A rapid filtration technique was used to study uptake of [^3^H]-MTX into MRP4 membrane vesicles in the presence of various concentrations of uremic toxins. Radioactivity was determined using liquid scintillation counting. Nonlinear regression analysis was performed using Graphpad Prism 5.02. Results are presented as mean ± SEM of one representative experiment performed in triplicate. Experiments were performed at least three times.

**Figure 4:**
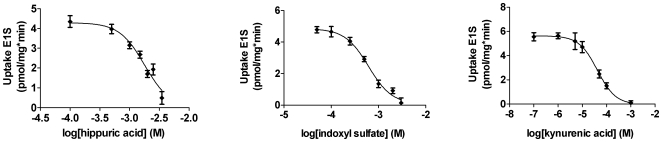
Uremic toxins inhibit BCRP-mediated transport. A rapid filtration technique was used to study uptake of [^3^H]-E1S into BCRP membrane vesicles in the presence of various concentrations of uremic toxins. Radioactivity was determined using liquid scintillation counting. Nonlinear regression analysis was performed using Graphpad Prism 5.02. Results are presented as mean ± SEM of one representative experiment performed in triplicate. Experiments were performed at least three times.

Using membrane vesicles overexpressing either transport protein, the interaction between ten uremic toxins and substrate specific transport by MRP4 and BCRP was studied. Hippuric acid dose-dependently inhibited MRP4-mediated [^3^H]-MTX uptake (Figure 3) and BCRP-mediated [^3^H]-E1S uptake (Figure 4) in a concentration range of 0.1 mM to 3.5 mM. The compound did not completely inhibit transport by MRP4, as depicted by the plateau at 50% of the inhibition curve, whereas uptake of [^3^H]-E1S by BCRP was completely blocked by hippuric acid. Indoxyl sulfate inhibited transport by both MRP4 and BCRP at concentrations ranging from 0.1 mM to 4 mM and 50 µM to 3 mM, respectively. Kynurenic acid inhibited substrate specific uptake by both MRP4 and BCRP in a concentration range of 0.1 µM to 1 mM. In addition, our results illustrate that indole-3-acetic acid and phenylacetic acid only reduced [^3^H]-MTX uptake by MRP4 both at concentrations ranging from 0.1 mM to 5 mM. Differences in initial substrate uptake arose from batch-to-batch variations of the membrane vesicles, however, all vesicles used demonstrated high substrate-specific uptake. The other toxins tested, *viz.* oxalate, p-cresol, p-toluenesulfonic acid, putrescine and quinolinic acid, did not decrease transport mediated by MRP4 or BCRP with more than 10% at a concentration of 1 mM, compared to control (Figure S1).

**Figure 5:**
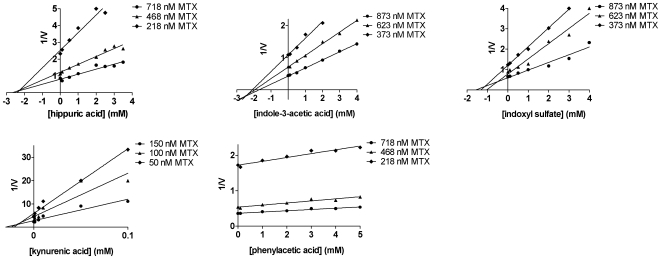
Uremic toxins inhibit MRP4-mediated transport mainly in a non-competitive fashion. A rapid filtration technique was used to study substrate specific uptake by MRP4 membrane vesicles in the presence of various concentrations of uremic toxins. Radioactivity was determined using liquid scintillation counting. Three independent dose-response experiments were performed, each in triplicate, using different concentrations of [^3^H]-MTX. A Dixon plot of the reciprocal of velocity was plotted against the concentration of different uremic toxins.

**Figure 6:**
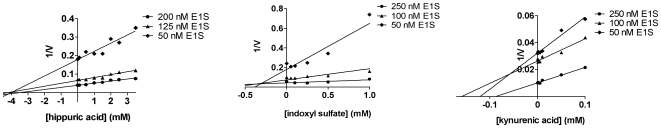
Uremic toxins inhibit BCRP-mediated transport mainly in a non-competitive fashion. A rapid filtration technique was used to study substrate specific uptake by BCRP membrane vesicles in the presence of various concentrations of uremic toxins. Radioactivity was determined using liquid scintillation counting. Three independent dose-response experiments were performed, each in triplicate, using different concentrations of [^3^H]-E1S. A Dixon plot of the reciprocal of velocity was plotted against the concentration of different uremic toxins.

To investigate the mode of interaction, the uptake of three different concentrations of substrate in the absence and presence of uremic toxin was studied. The resulting inhibition curves were transformed to a Dixon plot and analyzed by linear regression. As depicted in the Dixon plots, most curves intersect with the x-axis, indicating non-competitive inhibition. Figures 5 and 6 show that hippuric acid and indoxyl sulfate inhibited MRP4-mediated transport in a non-competitive manner, with a Ki of 25 µM and 1 mM, respectively. Both toxins also inhibited transport by BCRP in a non-competitive fashion with a Ki of 4 mM and 0.5 mM, respectively. Furthermore, our results indicated non-competitive inhibition of MRP4-mediated transport by indole-3-acetic acid (Ki: 2 mM) and kynurenic acid (Ki: 25 µM), while the latter compound acted as a mixed inhibitor for [^3^H]-E1S transport by BCRP. Mixed inhibition is considered to be composed of competitive and non-competitive inhibition. The mode of inhibition induced by phenylacetic acid, could not be elucidated due to incomplete inhibition of MRP4-mediated transport by this toxin. The kinetic analysis is summarized in Table 2.

**Table 2:** Transport inhibition occurs at clinically relevant concentrations.

MRP4						
**Uremic toxin**	**IC_50_ (µM**)	**IC_20_ (µM)**	**Ki (µM)**	**C_m_ (µM)**	**C_u_ (µM)**	**C_max_ (µM)**
kynurenic acid	8±1	1.5±0.3	25±2	1	-	50
hippuric acid	990±180	350±20	2500±50	160	1380	2631
phenylacetic acid	7100±1600	1600±170	-	18	3490^a^	7664^b^
indoxyl sulfate	1750±110	530±70	1000±90	129	211	940
indole-3-acetic acid	1795±8	570±30	2000±70	4	5	52

BCRP						
**Uremic toxin**	**IC_50_ (µM)**	**IC_20_ (µM)**	**Ki (µM)**	**C_m_ (µM)**	**C_u_ (µM)**	**C_max_ (µM)**
kynurenic acid	40±1	10±1	50^c^	1	-	50
hippuric acid	3670±170	690±70	4000±100	160	1380	2631
indoxyl sulfate	770±120	390±30	500	129	211	940

Values are shown as mean (C_m_; concentration determined in this study), highest mean/median (C_u_) and maximal uremic concentration (C_max_). Both C_u_ and C_max_ were obtained from www.uremic-toxins.org, unless stated otherwise. IC_50_ and IC_20_ values are shown as mean ± SEM of three separate experiments performed in triplicate.

a C_u_ obtained from literature [33].

b Hypothetical C_max_, calculated as C_max_  =  C_u_ + 2 SD, as previously described [19].

c Ki for competitive inhibition.

### Accumulation of uremic toxins in plasma

**Figure 7:**
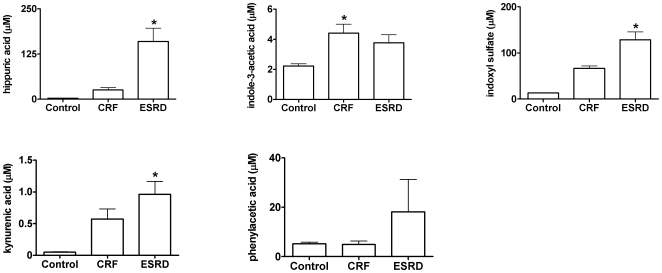
Accumulation of uremic toxins during CKD. HPLC was used to measure the total plasma concentrations of hippuric acid, indole-3-acetic acid, indoxyl sulfate and phenylacetic acid. LC/MS was used to measure kynurenic acid. Plasma samples were obtained from healthy volunteers (n = 4) and patients with CRF (n = 4) or ESRD (n = 6). Standards of the compounds were also analyzed in order to quantify the amount of toxins found in the samples. Acquired HPLC data were processed with PC1000 software (Spectrasystem) and LC/MS data were processed with Xcaliber software (Thermo scientific). Results are presented as mean ± SEM. *  =  p<0.05 compared to control.

To investigate whether the observed transport inhibition induced by several uremic toxins occurs at clinically relevant concentrations, the plasma levels of these toxins were measured in CRF and ESRD patients using HPLC and LC/MS-MS. Figure 7 illustrates that mean hippuric acid levels increased from 2.2 µM to 25 µM in CRF patients and to 160 µM in patients with ESRD. Furthermore, it was observed that indoxyl sulfate concentrations markedly increased from 13 µM (control) to 65 µM (CRF) and 129 µM (ESRD). The levels of indole-3-acetic acid significantly increased in patients with renal failure compared to control (2 µM), however, no differences were observed between patients with CRF (4 µM) or ESRD (4 µM). Furthermore, mean kynurenic acid levels significantly increased from 0.05 µM to 0.6 µM in CRF patients and to 1 µM in patients with ESRD. The mean plasma concentrations of phenylacetic acid slightly increased during CKD (CRF: 4 µM, ESRD: 18 µM), compared to control levels (5 µM), although not significantly. Hence, the interindividual variability increased with disease severity, with maximum concentrations reaching 9 µM in CRF patients and 83 µM in patients with ESRD.

### Inhibition of MRP4- and BCRP-mediated transport occurs at clinical relevant concentrations

The calculated Ki and IC_20_ values were compared with the mean plasma concentrations measured in ESRD patients during this study (C_m_), the highest mean/median (C_u_) and the highest maximal plasma concentrations (C_max_) measured in the literature in patients with CKD are also shown. The IC_20_ value was used since we believe that a 20% decrease in transport can already have a clinical impact. The C_u_ and C_max_ values were obtained from the online database of the European Uremic Toxin (EUTox) Work Group (www.uremic-toxins.org; [19]), unless stated otherwise. The results are summarized in Table 2. Kynurenic acid potently inhibited transport by MRP4, the Ki (25 µM) being notably lower than C_max_ (50 µM) and the IC_20_ (1.5 µM) similar to the mean concentration of this compound. Furthermore, our results indicated that 50% of MRP4-mediated uptake was inhibited by 2.5 mM hippuric acid, which is lower than the C_max_ of this solute (2.6 mM) and the IC_20_ (0.35 mM) is four times lower than the C_u_. Phenylacetic acid inhibited 50% of the transport by MRP4 at a concentration lower than the C_max_ (7 mM vs. 7.7 mM), whereas, 20% of the transport was inhibited at a concentration lower than the highest median plasma concentration (1.6 mM vs. 3.5 mM). Moreover, the Ki of indoxyl sulfate is almost similar to the C_max_ of this solute (1 mM vs. 940 µM), whereas, the IC_20_ (530 µM) is approximately two-times below the maximal concentration. Indole-3-acetic acid is the least potent inhibitor of MRP4-mediated transport, with both the Ki (2 mM) and the IC_20_ (570 µM) noticeably higher than the C_max_ (52 µM). BCRP-mediated transport is significantly inhibited by kynurenic acid, with the Ki (50 µM) comparable to the peak uremic toxin concentration (50 µM) and the IC_20_ (10 µM) lower than the C_max_. Indoxyl sulfate acid inhibited 50% of the uptake by BCRP at a concentration below the maximal uremic concentration (500 µM vs. 940 µM). Furthermore, hippuric acid inhibited 20% of the [^3^H]-E1S uptake by BCRP at a concentration almost two-fold lower than the highest median uremic concentration (690 µM vs. 1.4 mM), whereas the Ki (4 mM) was higher than the C_max_ (2.6 mM).

## Discussion

This study reports, for the first time, that several uremic toxins directly inhibit transport by two important efflux pumps, *viz.* MRP4 and BCRP, at clinically relevant concentrations. Since MRP4 and BCRP are located at the apical membrane of proximal tubule cells, transport activity depends on the intracellular levels of substrates rather than substrate concentrations in the blood. Previously, Masereeuw *et al.* demonstrated that methyl hippuric acids accumulate during secretory transport in the isolated perfused rat kidney [20], [21]. Furthermore, they showed that 2-methyl hippuric acid levels were 175-times higher in kidney tissue compared to the perfusate and 4-methyl hippuric acid concentrations were even 600-times higher. Thus, it is likely that intracellular uremic toxin concentrations are much higher than total plasma concentrations. This indicates that our results probably underestimate the potential inhibitory effect of uremic toxins on MRP4- and BCRP-mediated transport *in vivo.*

Both MRP4 and BCRP belong to the superfamily of ATP-binding cassette (ABC) transporters, a family of transmembrane proteins involved in the efflux of endo- and xenobiotics [22]. They are expressed in several tissues including liver, intestine, brain and the kidney [22], [23]. In addition to their contribution to the renal secretion of endogenous compounds, MRP4 and BCRP are involved in the extrusion of a broad range of drugs [22]. Despite their importance, little is known about the expression and activity of both MRP4 and BCRP during renal disease. Lu *et* al. showed, in a rat model of CKD, that BCRP gene expression decreased in correlation with disease severity in male rats. In contrast, both gene and protein expression of MRP4 remained unaltered [24]. Furthermore, it was demonstrated that following acute kidney injury in mice the gene expression of MRP4 increased whereas the expression of BCRP decreased. Conversely, protein expression of both transporters showed an opposite effect [25]. The impact of kidney disease on transporter expression and the inhibition of transport activity by uremic toxins, as described in this study, indicates that during CKD drug disposition may be altered leading to an increased risk of adverse drug reactions.

Other transporters of the ABC family are P-glycoprotein (P-gp) and MRP2. These transporters are also expressed in the apical membrane of proximal tubule cells, amongst other tissues, and, similar to MRP4 and BCRP, they are involved in the urinary excretion of drugs [22]. It was described that the activity of P-gp in rat kidneys decreased following glycerol-induced acute kidney injury, whereas, P-gp expression increased [26]. In addition, it was demonstrated in rats that CRF induced the expression of MRP2 in the kidney while P-gp expression remained unaltered [27]. Although P-gp and MRP2 have not been associated with uremic toxin clearance, these reports further support the hypothesis that pharmacokinetics can be altered during kidney disease due to alterations in expression and activity of transporters.

Competition for renal excretion by different compounds with similar structural characteristics has been demonstrated decades ago in canine models [28]. These classic experiments used *in vivo* models to investigate the effect of certain compounds on the renal clearance of an entity with a similar chemical structure. However, these models do not allow to distinguish between competitive or non-competitive inhibition, but merely showed an overall effect on renal clearance which includes various kinetic steps. Nowadays, by applying molecular tools such as the membrane vesicle transport assay, the interaction between substrates/inhibitors and a transporter of interest can be studied in detail without the interference of other transporters and metabolizing enzymes present in available *in vivo* and *in vitro* models. However, the membrane vesicle transport assay is based on the premise that *in vitro* uptake is similar to *in vivo* efflux. Nevertheless, the use of isolated membrane vesicles to study transport by efflux transporters has been proven to be suitable in both fundamental science and drug discovery [22]. The uremic toxins inhibit transport predominantly in a non-competitive fashion, suggesting that the toxins tested may use a different binding site than [^3^H]-MTX or [^3^H]-E1S for either MRP4 or BCRP, or that the toxins are not a substrate for the efflux pumps. It is known that MRP4 has multiple binding sites and simultaneously transports urate and cAMP or cGMP [10]. Moreover, there is also evidence suggesting that BCRP contains multiple binding regions [29], [30]. These findings suggest that the toxins tested may show different inhibition profiles when studied with other substrates for the transporters. Therefore, it is still possible that the tested uremic toxins are also substrates for MRP4 and BCRP. Evidently, more research is needed to fully elucidate the molecular interaction of uremic toxins with MRP4 and BCRP.

Our study further demonstrates that uremic toxins accumulate in patients with renal failure. We hypothesize that increasing MRP4- and BCRP-mediated transport activity is an important therapeutic target to prevent or reduce the accumulation of uremic toxins in dialysis patients. This hypothesis is supported by the study of Toyohara *et al.* who showed that overexpression of SLCO4C1 in the kidney reduces plasma levels of several uremic toxins in nephrectomized rats. Furthermore, they demonstrated that the transcription of SLCO4C1 is regulated by a xenobiotic responsive core element and that several statins induced the transcription of SLCO4C1 [7]. It would be interesting to examine whether statins, or drugs with a similar safety and tolerability profile, affect the expression and activity of MRP4 and BCRP.

In the present study the concentrations of five uremic toxins were measured in patients with CRF and ESRD. The CRF patients have severe renal insufficiency but were not yet on dialysis. The ESRD patients were treated with either peritoneal dialysis or hemodialysis. Plasma levels of urea and creatinine, substances not actively secreted to a considerable extent, were quite comparable between the groups. Still, our results indicate that the levels of several uremic toxins were higher in the ESRD patients as compared to the CRF patients. These results are in line with previous studies demonstrating that blood levels of indoxyl sulfate, p-cresol and uric acid are lower in patients with residual renal function [31], [32]. Thus, residual renal function importantly contributes to the clearance of uremic toxins, supporting the hypothesis that active transport is a necessity for the removal of uremic toxins.

Importantly, the mean concentrations of hippuric acid, indoxyl sulfate and phenylacetic acid reported in this study are lower than the mean/median concentrations in patients reported in literature [19], [33]. In contrast, the fold-increase of these toxins in ESRD patients compared to healthy controls, are higher in our study than in a preceding study [34]. Previously, Vanholder *et al*. noted that many discrepancies exist in the reported blood levels of uremic toxins in patients with renal failure [19]. These authors proposed several causes for these differences, including technical reasons (*e.g.* incomplete elution of compounds during chromatography or insufficient extraction of compounds from blood) and deviations in study population. Furthermore, it is important to notice that the largest variations were found in the concentrations of solutes derived from dietary intake. For example, hippuric acid is a metabolite of phenolic compounds found in tea, wine and fruit juices and phenylacetic acid is present in many fruits. Thus, it is likely that differences in diet between the study populations contribute largely to the observed variations in uremic toxin levels.

CKD is characterized by progressive and irreversible loss of renal function and the pathophysiological mechanisms underlying the progression of renal failure remain elusive. Concluding, several uremic toxins inhibit substrate-specific transport by MRP4 and BCRP at clinically relevant concentrations. Our results depict a novel pathway via which uremic toxins impede kidney excretory function and can contribute to accumulation of these potentially toxic uremic retention solutes.

## Supporting Information

Figure S1 **Several uremic toxins do not decrease BCRP- or MRP4-mediated transport.** A rapid filtration technique was used to study the ATP-dependent uptake of [^3^H]-MTX into MRP4- overexpressing membrane vesicles **(panel A)** and [^3^H]-E1S into BCRP membrane vesicles **(panel B)**. in the absence or presence of various uremic toxins (1 mM). Radioactivity was determined using liquid scintillation counting. Results are presented as mean ± SEM of one representative experiment performed in triplicate. Experiments were performed at least two times. *  =  p<0.01 compared to control. IAA, indole-3-acetic acid; Ox, oxalate; PC, p-cresol; PA, phenylacetic acid; PTA, p-toluensulfonic acid; Pu, putrescine; QA, quinolinic acid.
(TIF)

Table S1 **Results regression analysis.**
(DOC)
