# Supplementary figures and images for: Uremic Toxins Inhibit Transport by Breast Cancer Resistance Protein and Multidrug Resistance Protein 4 at Clinically Relevant Concentrations
Source: PLoS One. 2011 Apr 4;6(4):e18438. doi: 10.1371/journal.pone.0018438 (PMC3070735; doi:10.1371/journal.pone.0018438)

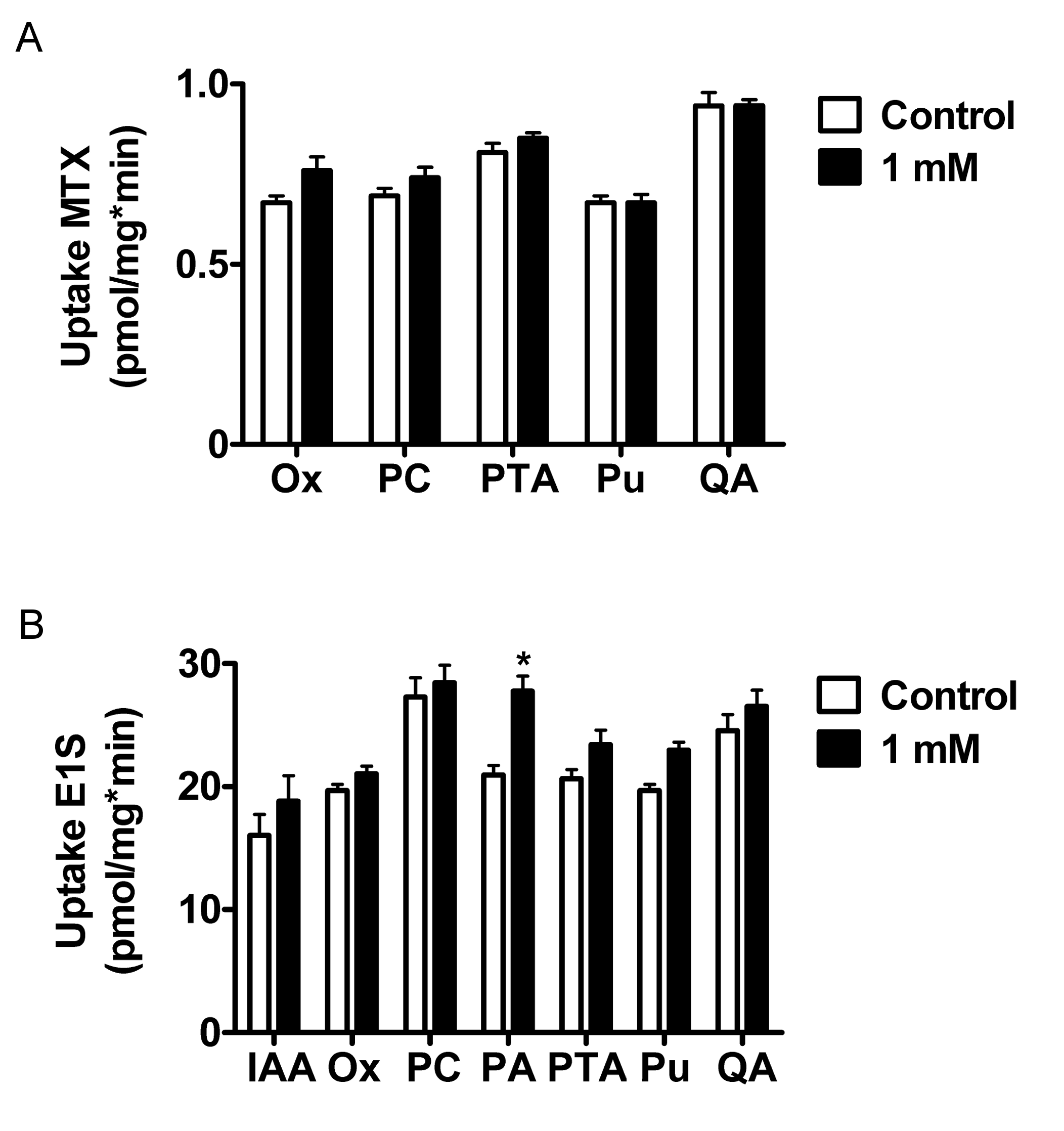

Supplement: Figure S1 — Several uremic toxins do not decrease BCRP- or MRP4-mediated transport. A rapid filtration technique was used to study the ATP-dependent uptake of [3H]-MTX into MRP4- overexpressing membrane vesicles (panel A) and [3H]-E1S into BCRP membrane vesicles (panel B). in the absence or presence of various uremic toxins (1 mM). Radioactivity was determined using liquid scintillation counting. Results are presented as mean ± SEM of one representative experiment performed in triplicate. Experiments were performed at least two times. * = p<0.01 compared to control. IAA, indole-3-acetic acid; Ox, oxalate; PC, p-cresol; PA, phenylacetic acid; PTA, p-toluensulfonic acid; Pu, putrescine; QA, quinolinic acid. (TIF) [file pone.0018438.s001.tif]
