# Supplementary material for: Uremic Toxins Inhibit Transport by Breast Cancer Resistance Protein and Multidrug Resistance Protein 4 at Clinically Relevant Concentrations
Source: PLoS One. 2011 Apr 4;6(4):e18438. doi: 10.1371/journal.pone.0018438 (PMC3070735; doi:10.1371/journal.pone.0018438)
Supplement: Table S1 — Results regression analysis. (DOC) [file pone.0018438.s002.doc]

| **Uremic toxin** | **transporter** | **Dose response curve** | | **Dixon plot** | | |
| --- | --- | --- | --- | --- | --- | --- |
|  |  | Constrained | Goodness of fit (R^2^) | Goodness of fit (r^2^) | Goodness of fit (r^2^) | Goodness of fit (r^2^) |
| **hippuric acid** | MRP4 | no | 0,9660 | 0,9274 | 0,9617 | 0,9337 |
| **indole-3-acetic acid** | MRP4 | yes | 0,9909 | 0,9985 | 0,9966 | 0,9766 |
| **indoxyl sulfate** | MRP4 | yes | 0,9744 | 0,9435 | 0,9685 | 0,9963 |
| **phenylacetic acid** | MRP4 | yes | 0,9511 | 0,9708 | 0,9410 | 0,9485 |
| **kynurenic acid** | MRP4 | no | 0,9582 | 0,9296 | 0,8399 | 0,9838 |
| **hippuric acid** | BCRP | yes | 0,9332 | 0,9661 | 0,9417 | 0,8749 |
| **indoxyl sulfate** | BCRP | no | 0,9801 | 0,6349 | 0,9355 | 0,9716 |
| **kynurenic acid** | BCRP | no | 0,9767 | 0,9887 | 0,9476 | 0,9701 |

**Table S1. Results regression analysis.**

Dose response curves were fitted using non-linear regression. For some curves the bottom was constrained equal to zero to enable accurate fitting. The goodness of fit was calculated from the sum of the squares of the distances of the points from the best-fit curve determined by non-linear regression (R^2^) or linear regression (r^2^).
